# Supplementary material for: ProteinShader: illustrative rendering of macromolecules
Source: BMC Struct Biol. 2009 Mar 30;9:19. doi: 10.1186/1472-6807-9-19 (PMC2672931; doi:10.1186/1472-6807-9-19)
Supplement: Additional file 1 — ProteinShader program without source code. This compressed file contains the complete ProteinShader program including associated libraries, but no source code. A README.txt file gives an overview of the ProteinShader distribution, and the index.html file in the help subdirectory has directions on getting started with the program as well as a set of tutorials. [file 1472-6807-9-19-S1.zip › ProteinShader-beta-0_9_4-binary/help/api/org/proteinshader/structure/class-use/AminoAcid.html]

Uses of Class org.proteinshader.structure.AminoAcid (ProteinShader API)


|  |  |  |
| --- | --- | --- |
| **Overview** \| **Package** \| **Class** \| **Use** \| **Tree** \| **Deprecated** \| **Index** \| **Help** | |  |
| PREV   NEXT | **FRAMES**    **NO FRAMES**     **All Classes** |  |


---


## **Uses of Class org.proteinshader.structure.AminoAcid**

| Packages that use AminoAcid | |
| --- | --- |
| **org.proteinshader.structure** | Holds the classes that store information from a Protein Data Bank file: Structure, Model, Chain, AminoAcid, Heterogen, Water, Atom, Bond, Helix, BetaStrand, Loop, *etc*. |
| **org.proteinshader.structure.visitor** | Holds Visitor classes that know how to traverse the hierarchy of objects held by class Structure. |

Uses of AminoAcid in org.proteinshader.structure

| Methods in org.proteinshader.structure that return AminoAcid | |
| --- | --- |
| `AminoAcid` | `Chain.addNewAminoAcid(AminoAcidEnum type, String residueID)`             Creates a new AminoAcid with the type and Residue ID given as arguments, adds the AminoAcid to the Chain's collection of AminoAcids, and returns a reference to the new AminoAcid. |
| `AminoAcid` | `Segment.getAminoAcid()`             Returns the a reference to the AminoAcid that this Segment corresponds to. |
| `AminoAcid` | `Chain.getAminoAcid(String residueID)`             Returns the AminoAcid with the residueID given as an argument. |
| `AminoAcid` | `Region.getAminoAcidAfterRegion()`             Returns the AminoAcid after this Region (or null if it does not exist). |
| `AminoAcid` | `Region.getAminoAcidBeforeRegion()`             Returns the AminoAcid before this Region (or null if it does not exist). |

| Methods in org.proteinshader.structure that return types with arguments of type AminoAcid | |
| --- | --- |
| `Iterator<AminoAcid>` | `Chain.iteratorAminoAcids()`             Returns an Iterator for the AminoAcids held by this Chain. |
| `Iterator<AminoAcid>` | `Region.iteratorAminoAcids()`             Returns an Iterator for the AminoAcids referred to by this Region. |

Constructors in org.proteinshader.structure with parameters of type AminoAcid

`Segment(AminoAcid aminoAcid,
Hermite hermite1,
Hermite hermite2,
Quaternion startRotation,
Quaternion middleRotation,
Quaternion endRotation,
boolean alwaysCapStart,
boolean alwaysCapEnd)`
  
          Creates a Segment.

Uses of AminoAcid in org.proteinshader.structure.visitor

| Methods in org.proteinshader.structure.visitor with parameters of type AminoAcid | |
| --- | --- |
| `void` | `FrenetFrameGeneratorVisitor.calculateRotation(AminoAcid prev, AminoAcid cur, AminoAcid next)`             Calculates the equivalent of a discrete Frenet frame (a rotation matrix [N B T]) and uses it to set the rotation attribute of the current AminoAcid. |
| `void` | `Visitor.visit(AminoAcid aminoAcid)`             Traverses all Atoms owned by the AminoAcid if AAPortionMode is set to ENTIRE\_AA. |
| `void` | `SFWriterVisitor.visit(AminoAcid aminoAcid)`             Writes all information held by an AminoAcid. |
| `void` | `FrenetFrameGeneratorVisitor.visit(AminoAcid aminoAcid)`             The AminoAcid being visited is considered as CA(i+1), while the last two AminoAcids are considered to be CA(i) and CA(i-1). |
| `void` | `LoopGeneratorVisitor.visit(AminoAcid aminoAcid)`             Checks whether a start AminoAcid and an end AminoAcid for a Loop have been found, and creates a new Loop as needed. |
| `void` | `AminoAcidLabelVisitor.visit(AminoAcid aminoAcid)`             Adds a residueID to the list of labels if it has not been seen before. |
| `void` | `BondGeneratorVisitor.visit(AminoAcid aminoAcid)`             Uses Atom distances to generate Bonds within the AminoAcid, and also uses the memory of the last carbonyl carbon seen to check for a possible peptide Bond. |

---


|  |  |  |
| --- | --- | --- |
| **Overview** \| **Package** \| **Class** \| **Use** \| **Tree** \| **Deprecated** \| **Index** \| **Help** | |  |
| PREV   NEXT | **FRAMES**    **NO FRAMES**     **All Classes** |  |


---

# *Copyright © 2007-2008*
